# The Grip of the Specialist

**Published:** 1907-10-12

**Authors:** 


### The Hospital

A Journal of

The Medical Sciences and Hospital Administration.

New Series. No. 29, Vol. II. [No. 1101, Vol. XLIII.] SATURDAY, OCTOBER 12, 1907.

### THE GRIP OF THE SPECIALIST.

Under this title, our lay contemporary, *The Nation*, published in one of its recent issues an article containing a number of grave reflections on some of the present customs and recent developments of medical practice. The fundamental basis of these reflections is to be found in the suggestion that the spread of specialism has led to an elaborate professional organisation, the various parts of which are connected by a common financial interest. The local general practitioner is "attached," we are told, to a "big" consulting physician in London; he, in turn, recommends a specialist, who "may propose" an operation and introduce the patient to a nursing home. Similarly the oculist "has" his optician, the specialist his instrument maker, and so, in due course and with appropriate relationship, are arranged the "chemist whose drugs are most reliable," the special sanatorium or seaside resort, and, for more wealthy patients, "a particular hotel and a friendly foreign doctor." In all this, it is said, there is a "network of community of interests" with a "system of profit-sharing and of commissions," and the whole arrangement is supported "by a legal procedure and a no less binding etiquette." The result has been the development of a series of inter-dependent "private profitable callings," as may be gathered from "occasional disclosures" regarding financial relations existing between physicians and surgeons on the one hand, and nursing homes, hotels, cures, and chemists on the other.

With all these decidedly offensive suggestions it is but fair to recognise that the writer of the article is by no means wanting in generous appreciation both of the scientific achievements of the profession and of the humanity of its individual members. He has no belief that any "conscious conspiracy" against the lives or purses of the public exists among medical practitioners, whose standard of personal honour he regards as at least as high as that of ordinary men. But he seems to be in genuine fear that existing conditions are too strong for us, and that with so many opportunities for ill-doing it is almost impossible to be more than "indifferent honest." Hence, he believes, it has become for himself and his fellow layman "a mere chance" whether, when taken ill, the treatment will be a dangerous operation, a novel and hazardous diet, two months' close confinement, or a long term of distant exile. Such a state of mental panic may well cause confusion of judgment, and we desire in all sincerity to say one or two words for the purpose of restoring the balance of common sense.

In the first place we cannot but regret that our contemporary has left the charges of profit-sharing and of the receipt of secret commissions so impersonal and vague. "Occasional" is hardly a term of precision, and so far as we can recollect disclosures of the nature suggested in the article have been most exceptional. Certainly we have no hesitation in saying that the public opinion of the profession is opposed in the strongest possible manner to anything in the nature of profit-sharing and percentages, and that any practitioner proved guilty of such actions would be vigorously condemned. If these practices exist, they are conducted in secrecy, and are confined to those whose sense of professional honour and obligation has suffered complete eclipse. It is quite true, as our critic alleges, that the practice of medicine may be made the opportunity for the financial aggrandisement of the practitioner at the expense of the patient. The ignorant, the superstitious, the foolish, and the credulous offer themselves as an easy prey, and the technicalities of practice may readily be used so as to mislead even the educated and intelligent. But all this places any man of common honour and honesty under a sense of special obligation. He has but two alternatives—the one to advise what he sincerely believes to be for the patient's benefit, and the other to stoop to a depth of baseness which would excite the contempt and disgust of a robust pick-pocket. Of course it is said that the departure from honest practice is an "unconscious" act, the necessary result of professional bias. But the gap is too wide to be thus bridged, and medical practitioners may claim to possess at least average intelligence. Hence we have no hesitation in repudiating both the suggestion of financial corruption and the charge of unconscious conspiracy. Personally we have never been inclined to take part in protestations of the "nobility" of medical practice. The practitioner must either act impartially in the interests of the patient or he must be guilty of the meanest type of fraud. To choose the one and reject the other is no more a cause of self-congratulation than is abstinence from crime in spite of opportunity. But, at the same time, we energetically repel the suggestion that, either wilfully or as a result of mental blindness, medical men are apt to confuse the two courses and to stumble inadvertently into a career of deceit and fraud.

The writer of the article in question tells us that dread of the practices he relates is spreading a feeling of grave, though impotent alarm through the community, and he is genuinely anxious to know how he may escape the terror of the professional network. We will tell him in a word. Let him select a competent and reputable family practitioner and act on the advice he receives. If on any occasion a special opinion is needed let him arrange to obtain this through his medical adviser. The dangerous specialist, in so far as he exists, would soon decline into insignificance if only the public would adopt this suggestion. In the meantime, our candid critic may console himself. The organised system which he fears does not exist, and all the other terrors which weigh so heavily on his soul may be readily avoided by a little common sense and frank dealing with his family medical attendant.

